# Femoral neck system vs. cannulated screws for Pauwels type III femoral neck fracture in non-elderly patients: a systematic review and meta-analysis

**DOI:** 10.3389/fsurg.2026.1784258

**Published:** 2026-03-31

**Authors:** Junlong Song, Chan Kang, Jeong-Kil Lee, Rongcan Liu, Huan Chen, Junsheng Zhang, Sangheok Lee, Long Zheng, Jung-Mo Hwang

**Affiliations:** 1 Department of Orthopedic Surgery, Chungnam National University Hospital, Chungnam National University School of Medicine, Daejeon, Republic of Korea; 2 Department of Orthopedic Surgery, Chungnam National University Sejong Hospital, Chungnam National University School of Medicine, Daejeon, Republic of Korea; 3 Department of Orthopedic Surgery, Yanbian University Hospital, Yanji, China

**Keywords:** cannulated screws, femoral neck fractures, femoral neck system, meta-analysis, non-elderly adults

## Abstract

**Background:**

The optimal fixation strategy for Pauwels type III femoral neck fractures in non-elderly adults remains controversial because of the high risk of postoperative complications. The femoral neck system (FNS) has been developed to improve angular and rotational stability, but its clinical advantages over cannulated screws (CS) in this unstable fracture pattern remain unclear. This study aimed to compare the safety and effectiveness of FNS and CS in non-elderly patients with Pauwels type III femoral neck fractures.

**Methods:**

A systematic search of PubMed, Embase, Web of Science, the Cochrane Library, Scopus, the Korean database (RISS), and Chinese databases (CNKI and Wanfang) was conducted to identify comparative studies evaluating FNS vs. CS in non-elderly adults with Pauwels type III femoral neck fractures. Fracture healing time, weight-bearing time, postoperative complications, and Harris Hip Score (HHS) were analyzed. Pooled results were expressed as mean differences (MD), standard mean differences (SMD), or odds ratios (OR) with 95% confidence intervals (CI). Meta-analyses were performed using RevMan 5.4.

**Results:**

A total of ten studies were included, with a collective count of 557 non-elderly patients suffering from Pauwels type III femoral neck fractures. Among these patients, 276 patients underwent FNS treatment, whereas 281 patients were treated with CS. The findings indicated that FNS performed significantly better than did CS in terms of fracture healing time [standard mean difference (SMD) = −0.90; 95% CI (−1.30, −0.49); *P* < 0.0001], weight-bearing time [SMD = −1.17; 95% CI (−1.61, −0.74); *P* < 0.00001], total complications [OR = 0.16; 95% CI (0.08, 0.33); *P* < 0.00001] and the Harris Hip Score (HHS) [MD = 2.07; 95% CI (1.16, 2.97); *P* < 0.00001]. Nonetheless, the intraoperative blood loss in the CS group was less than that in the FNS group [MD = 21.88; 95% CI (12.48, 31.29); *P* < 0.00001].

**Conclusion:**

Despite increased intraoperative blood loss, FNS was associated with faster fracture healing, earlier mobilization, fewer postoperative complications, and superior hip function compared with CS. These findings suggest that FNS may be a more effective and safer fixation option for Pauwels type III femoral neck fractures in non-elderly adults.

**Systematic Review Registration:**

https://www.crd.york.ac.uk/PROSPERO/view/CRD420251111561, PROSPERO CRD420251111561.

## Introduction

Femoral neck fractures account for approximately 50% of hip fractures globally, with projections indicating an increase of 6.3 million annual cases by 2050, and an increasing number of cases have also been reported among non-elderly individuals in recent years (1–3). Clinically, patients aged <65 years are commonly classified as a non-elderly population in the context of femoral neck fractures. Previous studies have shown that the outcomes of internal fixation in elderly patients aged ≥65 years are significantly worse than those in non-elderly patients aged <65 years (4). Complications such as nonunion (9.3%), femoral head necrosis (14.3%), and femoral neck shortening are prevalent in non-elderly patients, with 20% requiring revision surgery (5). Typically, Pauwels type III fractures of the femoral neck are more prevalent among non-elderly adults, and managing these fractures is often more challenging, resulting in a higher incidence of complications. Furthermore, as the Pauwels angle increases, the likelihood of experiencing postoperative complications also increases (6, 7). This is primarily attributed to the increased vertical shear forces generated at the fracture site as the Pauwels angle increases, which compromise biomechanical stability, reduce resistance to varus collapse, and predispose the fracture to fixation failure, nonunion, femoral neck shortening, and osteonecrosis of the femoral head. In recent years, significant mechanical research has focused on the femoral neck system (FNS) as a treatment option for femoral neck fractures, and a limited number of meta-analyses have been published on this topic. However, previous studies did not consistently distinguish between elderly and non-elderly patients. As elderly individuals frequently present with osteoporosis and compromised bone quality, pooling these populations may confound the assessment of implant stability and clinical outcomes. In addition, differences in injury mechanisms, baseline comorbidities, and healing potential between elderly and non-elderly patients may further influence fixation stability and postoperative outcomes. Therefore, systematic studies and meta-analysis on the efficacy of FNS for treating Pauwels type III femoral neck fractures in non-elderly patients are lacking. This gap in the current literature underscores a critical need for more rigorous investigations into the outcomes of this treatment method.

For patients who require preservation of their native femoral head and hip joint function, internal fixation surgery is the primary treatment option. Cannulated screws (CS) provide various benefits, such as limited trauma, decreased surgical time, and less impairment to the blood supply of the femoral neck. This method has been commonly utilized in clinical settings for numerous years and currently ranks among the most frequently employed internal fixation surgical methods. However, its shear resistance is weak, especially in Pauwels type III femoral neck fractures, which are common in non-elderly people, there are many complications (8–10). Complications such as fixation failure, varus collapse, femoral neck shortening, and nonunion may contribute to an increased need for revision surgery in patients treated with CS (11).

As a new generation of internal fixation, FNS can reduce rotational displacement and provide angular stability. The bolt's dynamic design enables it to move along the plate barrel, facilitating dynamic fixation at the fracture end. Current biomechanical studies have shown that the values of FNS in the 15 mm shortening cycle of the femoral neck and leg in the Pauwels type III fracture are significantly higher than those of CS (9). This suggests that FNS provides greater mechanical stability in the treatment of unstable femoral neck fractures (Pauwels type III) compared with CS. However, FNS may be associated with increased intraoperative blood loss and higher implant-related costs (15).

The primary aim of this research is to systematically evaluate and compare the rates of postoperative complications and the restoration of hip joint functionality in non-elderly patients who have sustained Pauwels type III femoral neck fractures. The comparative analysis will focus on two treatment approaches: the FNS and CS. By doing so, the findings of this study seek to provide clinicians with a solid evidence-based foundation that can inform their decision-making processes in the management of these specific types of fractures. This research aspires to contribute valuable insights that could enhance patient care and outcomes in this area of orthopedic practice.

## Materials and methods

This research was performed in compliance with the Preferred Reporting Items for Systematic Reviews and Meta-Analyses (PRISMA) guidelines (12). This study plan has been officially registered to PROSPERO (registration number: CRD420251111561).

### Search strategy

A systematic search was performed in PubMed, Embase, Web of Science, Cochrane Library, Scopus, Korean database (RISS) and Chinese databases (CNKI, Wanfang) up to June 30, 2025. Keywords included “femoral neck fractures,” “femoral neck system” and “bone screws”.

### Eligibility criteria

The inclusion criteria: (1) patients aged 18–65 years with unilateral Pauwels III femoral neck fractures; (2) comparative studies (FNS vs. CS) with ≥ 6 months of follow-up; (3) reported outcomes: HHS, complications, fracture healing time, etc.; (4) study designs: RCTs or retrospective cohorts; For late complications (nonunion and femoral head necrosis), only studies with a mean follow-up duration of ≥12 months were included in the corresponding meta-analyses.

The exclusion criteria: (1) reviews, biomechanical studies, case reports, or incomplete data; (2) open or pathological fractures.

### Data extraction and quality assessment

Two reviewers independently extracted data, including study characteristics (author, year, sample size, and follow-up duration), interventions, and outcome measures such as operative time, intraoperative blood loss, fracture healing time, time to weight bearing, and postoperative complications, including osteonecrosis of the femoral head, nonunion, femoral neck shortening, and fixation failure. The criteria for weight-bearing progression were determined according to each individual study protocol and were generally based on clinical and radiographic assessment of fracture stability and healing. In cases where the investigators encountered inconsistencies in data extraction, a consensus was reached through a discussion.

The methodological index for non-randomized studies (MINORS) (13) scoring criteria was utilized to evaluate the quality of the study in this meta-analysis. The quality assessment was carried out by two independent reviewers, and any differences were addressed by discussing them with a third reviewer.

### Statistical analysis

The findings of the study were examined utilizing the Review Manager (RevMan) [Computer program. Version 5.4, The Cochrane Collaboration, 2020]. For continuous variables, a 95% confidence interval (CI) was employed, and the mean difference (MD) was selected as the measure of effect size when the units of measurement were consistent across studies. If the unit of measurement of the data is inconsistent, it is expressed by the standard mean difference (SMD). For dichotomous variables such as postoperative complications, the 95% CI and odds ratio (OR) were used for representation. And the heterogeneity was evaluated using the *I*^2^ statistic and *Q* test. The results of all included studies for the FNS group and the CS group were depicted using forest plots. When *P* ≤ 0.10 and *I*^2^ ≥ 50%, it indicates high heterogeneity, and a random-effects model was employed. If *P* > 0.10 and *I*^2^ < 50%, this indicates low heterogeneity, leading to the use of a fixed-effects model. If significant heterogeneity (*I*^2^ ≥ 50%) is determined, sensitivity analysis will be used. And sequentially excluding individual studies to identify outliers influencing heterogeneity. Publication bias was not formally assessed due to the limited number of included studies per outcome and the predominance of retrospective cohort designs (14).

## Results

### Included study

**Figure 1:**
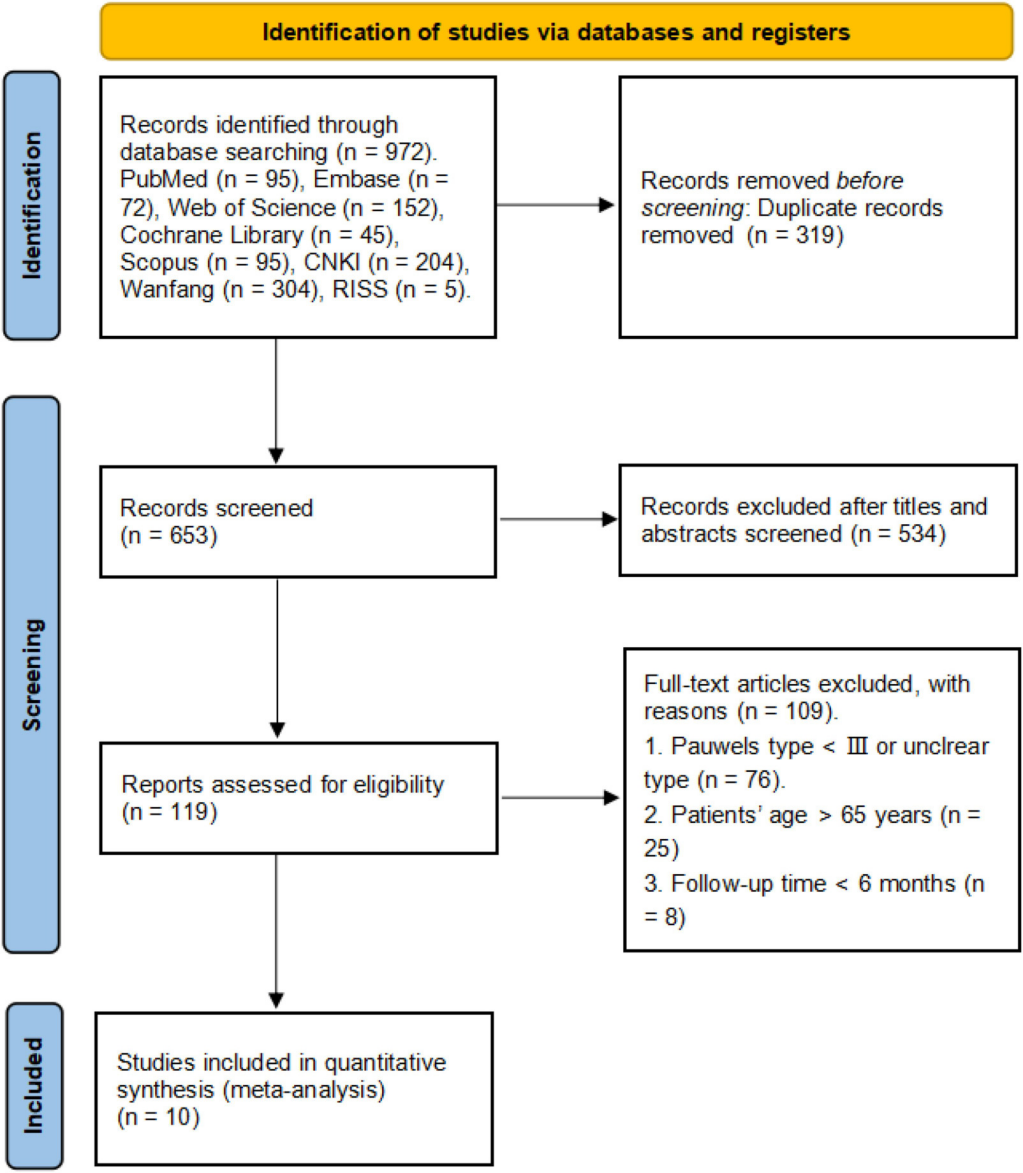
PRISMA 2020 flow diagram.

A preliminary search of electronic databases yielded 972 relevant articles, and 309 articles were obtained after removing duplicate articles published by the same author. A thorough examination of the titles and abstracts led to the exclusion of 534 articles that were deemed irrelevant to the aims of this study. This meticulous process ensured that only pertinent literature was considered. Subsequently, the full texts of the remaining articles were reviewed, and by employing specific inclusion and exclusion criteria, a final selection of ten articles (15–24) that fulfilled the requirements of the study was identified. To provide a clear visual representation of this meticulous literature retrieval process, a flow diagram has been included in Figure 1.

### Study characteristics

The ten studies analyzed were retrospective cohort studies encompassing a total of 557 individuals suffering from femoral neck fractures. A total of 276 patients received FNS, and 281 patients were administered CS therapy. Every participant enrolled in the research was diagnosed with a Pauwels type III fracture of the femoral neck, and the follow-up time was at least 6 months. The detailed information of these ten articles is listed in Table 1.

**Table 1:** Characteristics of included studies.

Study	Year	Group	Number of cases	Age (year)	Male/Female	From injury to surgery	Follow-up time (month)
Zhou et al. (15)	2021	FNS	30	54.53 ± 6.71	12/18	<48 (h)	10–22
		CS	30	53.14 ± 7.19	12/18		
Weng et al. (16)	2022	FNS	26	39.22 ± 6.48	17/9	3.72 ± 1.32 (d)	14 ± 2.29
		CS	26	42.31 ± 5.69	15/11	3.42 ± 1.04 (d)	
Wu et al. (17)	2022	FNS	20	41.95 ± 12.63	10/10	35.0 ± 7.3 (h)	>6
		CS	20	43.55 ± 10.10	9/11	33.0 ± 6.2 (h)	
Huang et al. (18)	2023	FNS	42	47.3 ± 6.8	18/24	34.8 ± 5.8 (h)	>12
		CS	45	49.1 ± 7.5	20/25	36.4 ± 6.4 (h)	
Wang et al. (19)	2023	FNS	33	46.5 ± 8.1	22/11	4.0 ± 0.8 (d)	14.27 ± 1.23
		CS	29	46.3 ± 7.6	19/10	3.7 ± 0.8 (d)	14.69 ± 1.58
Yuan et al. (20)	2023	FNS	18	52.4 (42–64)	7/11	40.5 (h)	9–17
		CS	28	49.7 (41–61)	9/19	44.2 (h)	
Zhang et al. (21)	2023	FNS	25	45.20 ± 11.88	14/11	NR	15.04 ± 3.88
		CS	27	49.30 ± 9.24	11/16		16.19 ± 5.35
Zhu et al. (22)	2024	FNS	54	43.1 ± 7.8	28/26	NR	11.2 ± 3.1
		CS	49	42.2 ± 8.2	26/23		11.5 ± 2.4
Ding et al. (23)	2025	FNS	11	52.55 ± 10.51	4/7	2.0 ± 0.6 (d)	>6
		CS	8	57.75 ± 4.80	5/3	2.0 ± 0.5 (d)	
Zhang et al. (24)	2025	FNS	17	47.65 ± 14.39	6/11	NR	11–24
		CS	19	49.26 ± 11.46	11/8		

NR, not reported; h, hour; d, day.

### Risk of bias assessment

All studies were deducted points because they were retrospective and did not use single blind or double-blind methods. All included studies had at least 19 points based on the MINORS score, indicating that the quality of the evidence was good, as detailed in Table 2.

**Table 2:** Risk of bias assessment.

Study	A clearly stated aim	Inclusion of consecutive patients	Prospective collection of data	Endpoints appropriate to the aim of the study	Unbiased assessment of the study endpoint	Follow-up period appropriate to the aim of the study	Loss to follow up less than 5%	Prospective calculation of the study size	An adequate control group	Contemporary groups	Baseline equivalence of groups	Adequate statistical analyses	Total score
Zhou et al. (15)	2	2	2	2	0	1	2	0	2	2	2	2	19
Weng et al. (16)	2	2	2	2	0	2	2	0	2	2	2	2	20
Wu et al. (17)	2	2	2	1	0	2	2	0	2	2	2	2	19
Huang et al. (18)	2	2	2	2	0	2	2	0	2	2	2	2	20
Wang et al. (19)	2	2	2	2	0	2	2	0	2	2	2	2	20
Yuan et al. (20)	2	2	2	2	0	1	2	0	2	2	2	2	19
Zhang et al. (21)	2	2	2	1	0	2	2	0	2	2	2	2	19
Zhu et al. (22)	2	2	2	2	0	2	2	0	2	2	2	2	20
Ding et al. (23)	2	2	2	1	0	2	2	0	2	2	2	2	19
Zhang et al. (24)	2	2	2	2	0	2	2	0	2	2	2	2	20

## Meta-analysis results

### Intraoperative blood loss

**Figure 2:**
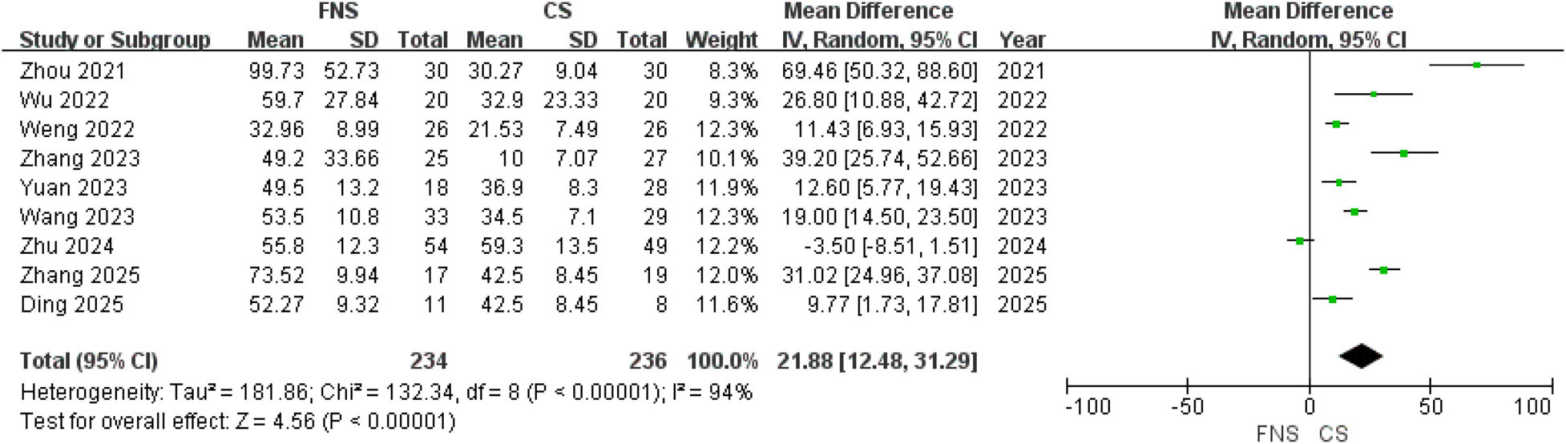
Forest plot comparing blood loss between the FNS and CS group.

Nine studies (15–17, 19–24) compared intraoperative blood loss between the two surgical modalities. The findings from the analysis showed that the blood loss during the operation was less in the CS group compared to the FNS group [MD = 21.88; 95% CI (12.48, 31.29); *P* < 0.00001] (Figure 2).

### Postoperative indicators

**Figure 3:**
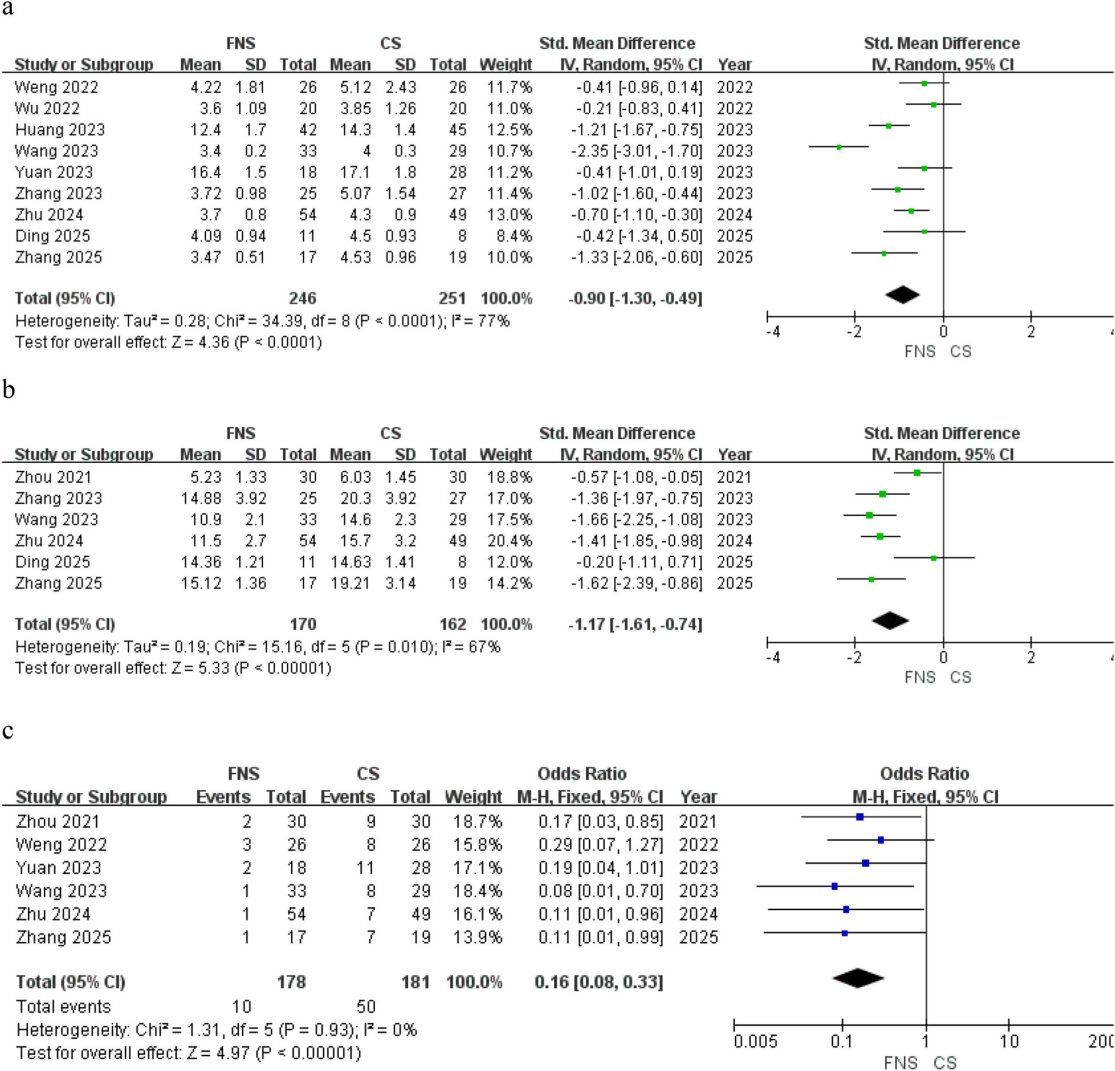
Forest plots comparing FNS and CS group. **(a)** Fracture healing time; **(b)** weight-bearing time; **(c)** total complication.

Postoperative indicators included fracture healing time, weight-bearing time, and the occurrence of total complications. Nine studies (16–24) reported the fracture healing time, and the findings revealed that the FNS group demonstrated significantly shorter fracture healing time compared with the CS group [SMD = −0.90; 95% CI (−1.30, −0.49); *P* < 0.0001] (Figure 3A). The analysis results from six studies (15, 19, 21–24) revealed that the postoperative weight-bearing time for patients in the FNS group was earlier than that for patients in the CS group [SMD = −1.17; 95% CI (−1.61, −0.74); *P* < 0.00001] (Figure 3B). Total complications were reported in six studies (15, 16, 19, 20, 22, 24), with 10 patients in the FNS group and 50 patients in the CS group. Upon analysis, the complication rate in the FNS group was found to be 5.6%, whereas in the CS group, it was 27.6%. The overall complication rate observed in the FNS group was significantly reduced in comparison to the rate recorded in the CS group [OR = 0.16; 95% CI (0.08, 0.33); *P* < 0.00001] (Figure 3C).

### Postoperative complications

**Figure 4:**
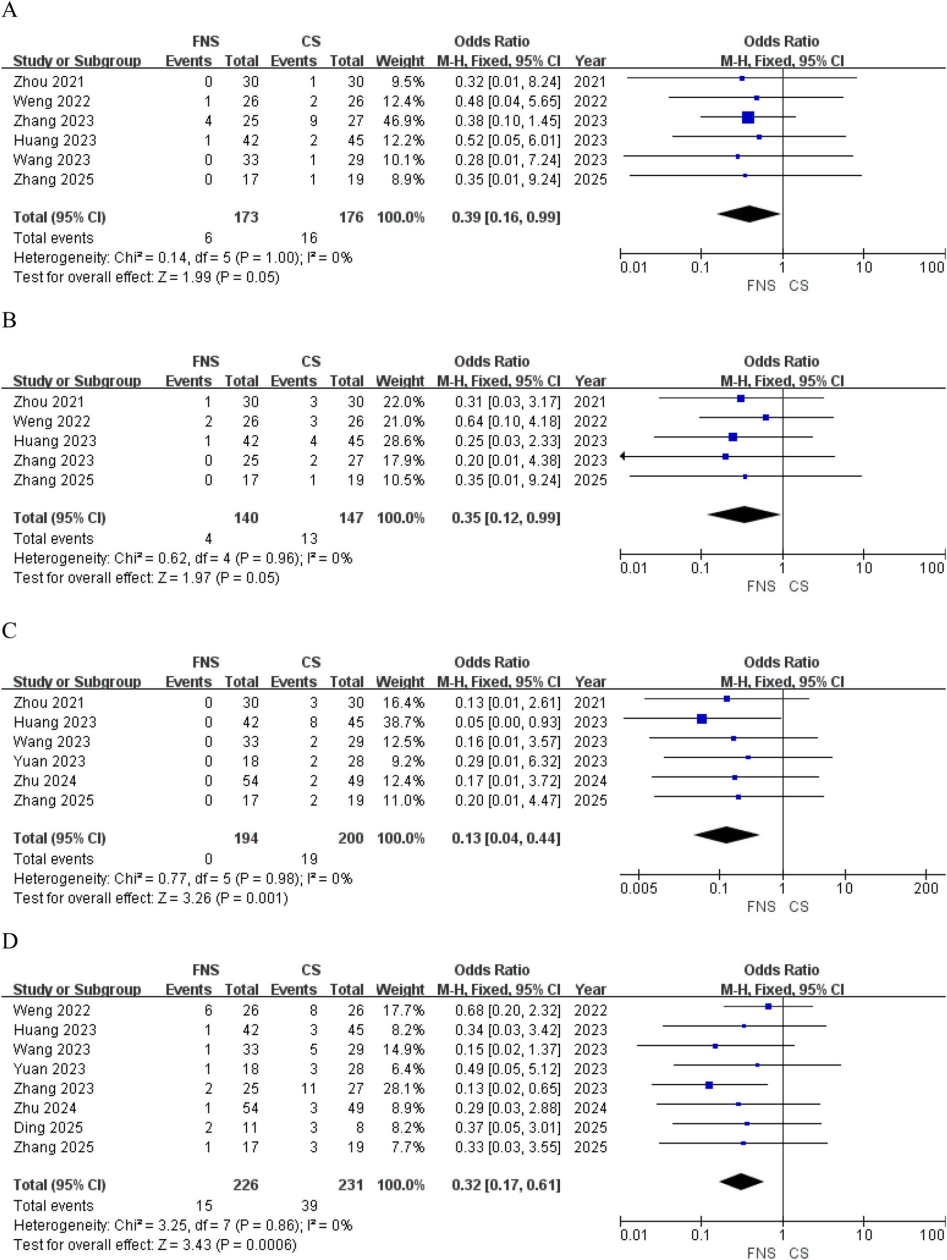
Forest plots comparing postoperative complications between the FNS and CS group. **(A)** Femoral head necrosis; **(B)** nonunion; **(C)** internal fixation failure; **(D)** femoral neck shortening.

Postoperative complications mainly included femoral head necrosis, fracture nonunion, internal fixation failure and femoral neck shortening. For late complications (femoral head necrosis and nonunion), only studies with a mean follow-up duration of ≥12 months were included in the pooled analyses. Six studies (15, 16, 18, 19, 21, 24) compared the incidence of postoperative femoral head necrosis. The pooled results showed a lower incidence of osteonecrosis in the FNS group than in the CS group, reaching borderline statistical significance [OR = 0.39; 95% CI (0.16, 0.99); *P* = 0.05] (Figure 4A). Pooled analysis of five studies (15, 16, 18, 21, 24) demonstrated a lower incidence of nonunion in the FNS group compared with the CS group, with borderline statistical significance [OR = 0.35; 95% CI (0.12, 0.99); *P* = 0.05] (Figure 4B). No significant heterogeneity was observed across studies [*I*^2^ = 0%, *P* = 0.96]. Six separate investigations (15, 18–20, 22, 24) indicated that internal fixation failure occurred post-surgery, with observations noting that the rate of internal fixation failure in the FNS group was lower compared to the CS group [OR = 0.13; 95% CI (0.04, 0.44); *P* = 0.001] (Figure 4C). Eight studies (16, 18–24) provided data on postoperative shortening of the femoral neck. Patients who underwent FNS therapy exhibited a lower frequency of femoral neck shortening compared to those who received CS treatment [OR = 0.32; 95% CI (0.17, 0.61); *P* = 0.0006] (Figure 4D). Detailed raw data of postoperative complications for each included study are provided in Supplementary Table S1.

### Postoperative scores

**Figure 5:**
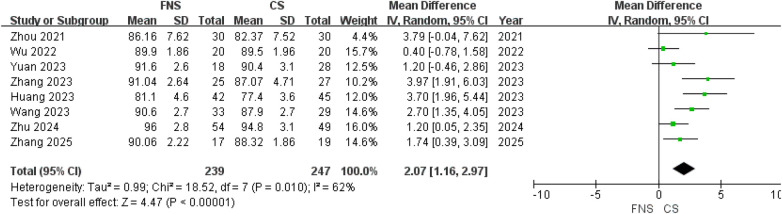
Forest plot comparing HHS at the the final follow-up between the FNS and CS groups.

Eight studies (15, 17–22, 24) have evaluated post-surgical restoration of joint functionality using HHS. Significantly higher HHS were observed in the FNS group than in the CS group at the final follow-up [MD = 2.07; 95% CI (1.16, 2.97); *P* < 0.00001] (Figure 5).

### Sensitivity analysis

Sensitivity analysis was conducted by sequentially removing individual studies to evaluate the results robustness. Upon exclusion of Wang et al.'s study (19), heterogeneity in fracture healing time substantially decreased (*I*^2^ = 47%, *P* < 0.00001). Similarly, removal of Zhou et al.'s research (15) significantly reduced heterogeneity in weight-bearing time (*I*^2^ = 48%, *P* < 0.00001). Elimination of Wu et al.'s data (17) likewise lowered heterogeneity in Harris Hip Scores (*I*^2^ = 48%, *P* < 0.00001). Sequential removal of individual studies did not substantially alter heterogeneity estimates for intraoperative blood loss, total complications, femoral head necrosis, nonunion, internal fixation failure, or femoral neck shortening, indicating robust results across all primary outcomes.

## Discussion

While CS remains widely used for femoral neck fractures due to its minimally invasive nature and procedural efficiency, emerging studies highlight its limitations in preventing complications. As an innovative stabilization approach, FNS combines angular stability (via plate-screw design) and rotational resistance, offering superior biomechanical strength compared to CS and DHS in unstable Pauwels III fractures (9). To our knowledge, this is the first meta-analysis focusing exclusively on Pauwels type III femoral neck fractures in non-elderly adults. Previous meta-analyses pooled stable and unstable fracture patterns together, which may obscure clinically meaningful differences. By restricting our analysis to high-shear, unstable fractures, our findings provide more specific and clinically relevant evidence for implant selection in this challenging patient population.

This meta-analysis compared FNS and CS in the treatment of Pauwels type III femoral neck fractures in non-elderly adults. This study revealed significantly greater intraoperative blood loss with FNS than with CS, although substantial heterogeneity was observed. Sensitivity analysis confirmed persistently high heterogeneity, indicating that this variability was not driven by any single study and supporting the robustness of the results. Heterogeneity may be attributed to differences in fracture reduction quality, implant positioning, surgeon experience, postoperative rehabilitation protocols, and follow-up duration.

The evaluation of the safety and effectiveness of internal fixation mainly depends on these aspects. The first is the healing time of the fracture, the second is the development of surgical complications, and the third is postoperative hip functional recovery, particularly in terms of range of motion, pain relief, and ambulatory capacity, is routinely assessed using validated scoring systems such as the Harris Hip Score (HHS). FNS accelerated fracture healing and reduced femoral neck shortening compared to CS, likely due to its dynamic compression mechanism and shear resistance (8, 9). Consequently, the typical duration for fracture healing among individuals in the FNS group is expected to be shorter than that experienced by those in the CS group, allowing patients to walk with weight earlier. The results of Lu et al.'s (25) study indicated no significant difference in fracture healing time between the two groups, which is contrary to the findings of our research. This discrepancy may be attributed to the fact that previous studies did not classify fracture types in detail and predominantly included patients with stable fractures, resulting in no significant differences in fracture healing time. However, our study specifically enrolled patients with unstable fractures, which demonstrated a greater incidence of complications prior to fracture union, thus leading to inconsistent findings compared with those reported by Lu et al. This meta-analysis suggests that FNS was associated with shorter fracture healing time and time to weight bearing in non-elderly patients. Earlier mobilization may help reduce complications associated with prolonged bed rest, although these outcomes were not directly evaluated in the present study. Future research is needed to explore the impact of implant selection on functional recovery and healthcare resource utilization.

Complications that occur after surgery play a significant role in assessing the safety and efficacy of internal fixation. Research by Ramadanov et al. (10, 11) demonstrated that while CS offers advantages in terms of intraoperative blood loss, it is associated with the highest reoperation risk compared with DHS and arthroplasty. Furthermore, their findings revealed that the risk of procedure-specific complications in CS-treated femoral neck fracture patients increases with advancing age, particularly in patients over 80 years old. This is consistent with our viewpoint that internal fixation is more suitable for non-elderly patients. The findings of this meta-analysis indicate that the overall complication rate associated with FNS is considerably lower compared to that of CS, particularly regarding femoral neck shortening and failure of internal fixation. This result further confirms the previous biomechanical experimental results (9), that is, FNS has more advantages in angular stability and rotational stability than CS. It is generally believed that proper compression between fractures is conducive to fracture healing, but when the femoral neck shortening is greater than 10 mm, it will seriously affect fracture healing and functional recovery (26). In a meta-analysis of complications of femoral neck fracture, it was shown that the total pooled incidence of avascular necrosis and nonunion was the highest, 14.3% and 9.3%, respectively (5). Our analysis showed lower rates of postoperative complications in the FNS group compared with the CS group, particularly for femoral neck shortening and internal fixation failure. The pooled estimates for osteonecrosis of the femoral head and nonunion also favored FNS, reaching borderline statistical significance. These findings are consistent with those reported by Jiang et al. (27) and further substantiate the superior safety profile of FNS in reducing postoperative complications.

The most commonly used method to evaluate hip function is the HHS, which mainly includes four parts: pain, function, deformity, and range of motion. This meta-analysis aimed to evaluate HHS at the final follow-up and demonstrated that patients who underwent FNS surgery exhibited a considerably higher HHS compared to those in the CS group. The superior clinical outcomes observed with FNS may be explained by its unique biomechanical design. The angular-stable plate–bolt construct enhances resistance to vertical shear forces, while the dynamic sliding mechanism allows controlled compression at the fracture site. These features likely contribute to faster fracture healing, reduced femoral neck shortening, and lower rates of fixation failure compared with CS, which primarily relies on frictional stability and is more susceptible to varus collapse in vertically oriented fractures.

Although FNS is superior to CS in many aspects, this study also has the following limitations. First, all included studies were retrospective cohort studies, which may introduce selection bias related to surgeon preference and institutional protocols. Second, the number of available studies and overall sample size remain limited, and high-quality randomized controlled trials are lacking. Third, heterogeneity across studies may arise from variations in surgical technique, reduction quality, rehabilitation protocols, and follow-up duration. Although we restricted late complication analyses to studies with a mean follow-up of at least 12 months, we acknowledge that longer-term follow-up (≥24 months) would provide stronger evidence, and currently available comparative studies remain limited. Finally, as most included studies were conducted in East Asia, the findings may not be fully generalizable to populations with different demographic characteristics, healthcare systems, and clinical practices.

### Clinical implications

Our findings support FNS as an effective fixation option for non-elderly adults with Pauwels type III femoral neck fractures, given its advantages in reducing postoperative complications, accelerating fracture healing, enabling earlier weight bearing, and improving hip function. Although greater intraoperative blood loss was observed with FNS, the overall clinical outcomes favored its use. In particular, FNS may be especially advantageous in high-shear fracture patterns such as Pauwels type III fractures, where enhanced angular and rotational stability is critical. Implant selection should ultimately be individualized based on fracture characteristics and clinical judgment.

## Conclusion

Despite greater intraoperative blood loss, the femoral neck system was associated with significantly fewer postoperative complications, faster fracture healing, earlier weight-bearing, and superior hip function compared with cannulated screws. These findings suggest that FNS may be the preferred fixation method for Pauwels type III femoral neck fractures in non-elderly adults. Prospective randomized trials are needed to further validate these outcomes.

## Data Availability

The original contributions presented in the study are included in the article/Supplementary Material, further inquiries can be directed to the corresponding author/s.
