# Supplementary material for: Femoral neck system vs. cannulated screws for Pauwels type III femoral neck fracture in non-elderly patients: a systematic review and meta-analysis
Source: Front Surg. 2026 Mar 31;13:1784258. doi: 10.3389/fsurg.2026.1784258 (PMC13076335; doi:10.3389/fsurg.2026.1784258)
Supplement: Supplementary file 1 [file Table1.docx]

**Supplementary Table S1** Detailed postoperative complication events in included studies

| Study | n(FNS) | ONFH(n) | nonunion (n) | fixation failure(n) | femoral neck shortening(n) | n(CS) | ONFH(n) | nonunion (n) | fixation failure(n) | femoral neck shortening(n) |
| --- | --- | --- | --- | --- | --- | --- | --- | --- | --- | --- |
| Zhou 2021 | 30 | 1 | 0 | 0 | NR | 30 | 1 | 3 | 3 | NR |
| Weng 2022 | 26 | 1 | 2 | NR | 6 | 26 | 2 | 3 | NR | 8 |
| Huang 2023 | 42 | 1 | 1 | 0 | 1 | 45 | 2 | 4 | 8 | 3 |
| Wang 2023 | 33 | 0 | NR | 0 | 1 | 29 | 1 | NR | 2 | 5 |
| Yuan 2023 | 18 | NR | NR | 0 | 1 | 28 | NR | NR | 2 | 3 |
| Zhang 2023 | 25 | 4 | 0 | NR | 2 | 27 | 9 | 2 | NR | 11 |
| Zhu 2024 | 54 | NR | NR | 0 | 1 | 49 | NR | NR | 2 | 3 |
| Ding 2025 | 11 | NR | NR | NR | 2 | 8 | NR | NR | NR | 3 |
| Zhang 2025 | 17 | 0 | 0 | 0 | 1 | 19 | 1 | 1 | 2 | 3 |

NR, not reported; ONFH, osteonecrosis of the femoral head.
